# Sexually dimorphic pubertal development and adipose tissue kisspeptin dysregulation in the obese and preeclamptic-like BPH/5 mouse model offspring

**DOI:** 10.3389/fphys.2023.1070426

**Published:** 2023-03-23

**Authors:** Viviane C. L. Gomes, Kalie F. Beckers, Kassandra R. Crissman, Camille A. Landry, Juliet P. Flanagan, Reham M. Awad, Fabio Del Piero, Chin-Chi Liu, Jenny L. Sones

**Affiliations:** ^1^ Department of Veterinary Clinical Sciences, Louisiana State University School of Veterinary Medicine, Baton Rouge, LA, United States; ^2^ Department of Pathobiological Sciences, Louisiana State University School of Veterinary Medicine, Baton Rouge, LA, United States

**Keywords:** puberty, testosterone, *Kiss1/Kiss1r*, obesity, adiposity

## Abstract

Preeclampsia (PE) is a devastating hypertensive disorder of pregnancy closely linked to obesity. Long-term adverse outcomes may occur in offspring from preeclamptic pregnancies. Accordingly, sex-specific changes in pubertal development have been described in children from preeclamptic women, but the underlying mechanisms remain vastly unexplored. Features of PE are spontaneously recapitulated by the blood pressure high subline 5 (BPH/5) mouse model, including obesity and dyslipidemia in females before and throughout pregnancy, superimposed hypertension from late gestation to parturition and fetal growth restriction. A sexually dimorphic cardiometabolic phenotype has been described in BPH/5 offspring: while females are hyperphagic, hyperleptinemic, and overweight, with increased reproductive white adipose tissue (rWAT), males have similar food intake, serum leptin concentration, body weight and rWAT mass as controls. Herein, pubertal development and adiposity were further investigated in BPH/5 progeny. Precocious onset of puberty occurs in BPH/5 females, but not in male offspring. When reaching adulthood, the obese BPH/5 females display hypoestrogenism and hyperandrogenism. Kisspeptins, a family of peptides closely linked to reproduction and metabolism, have been previously shown to induce lipolysis and inhibit adipogenesis. Interestingly, expression of kisspeptins (Kiss1) and their cognate receptor (Kiss1r) in the adipose tissue seem to be modulated by the sex steroid hormone milieu. To further understand the metabolic-reproductive crosstalk in the BPH/5 offspring, *Kiss1/Kiss1r* expression in male and female rWAT were investigated. Downregulation of *Kiss1/Kiss1r* occurs in BPH/5 females when compared to males. Interestingly, dietary weight loss attenuated circulating testosterone concentration and rWAT *Kiss1* downregulation in BPH/5 females. Altogether, the studies demonstrate reproductive abnormalities in offspring gestated in a PE-like uterus, which appear to be closely associated to the sexually dimorphic metabolic phenotype of the BPH/5 mouse model.

## 1 Introduction

Preeclampsia, a leading cause of maternal and fetal morbidity and mortality, is a hypertensive disorder of pregnancy characterized by new-onset hypertension after 20 weeks of gestation (systolic blood pressure >140 mmHg and diastolic blood pressure >90 mmHg), and clinical signs secondary to either hematopoietic, renal, hepatic, pulmonary, or neurological compromising (Burton et al., 2019; ACOG, 2020). The incidence of severe preeclampsia in the United States has increased approximately 6.7-fold over the course of 3 decades (Annath et al., 2013). Correspondingly, the prevalence of obesity, a major risk factor for preeclampsia, has also increased globally in the same period, with a substantial rise in the population of overweight children and adolescents (Ng et al., 2014; Olson et al., 2019). Importantly, transgenerational effects of maternal obesity and preeclampsia have been gradually unraveled, including long-term cardiovascular, metabolic, neurological and endocrine dysfunctions in offspring gestated in an obesogenic and hypertensive uterine environment (Wu et al., 2017; Fox et al., 2019; Lu and Hu, 2019).

While many investigations have focused on mechanisms underlying the risk of adverse cardiometabolic consequences in offspring from obese and preeclamptic mothers, the potential long-term reproductive outcomes remain largely unexplored (Wu et al., 2017; Fox et al., 2019; Lu and Hu, 2019). Sex-specific abnormalities in pubertal development may occur in children born from preeclamptic pregnancies (Ogland et al., 2011; Alsnes et al., 2016). In a population-based study, increased body mass index (BMI) and altered progression of pubertal development were described in daughters from preeclamptic pregnancies, with the latter being positively associated with the severity of maternal disease (Ogland et al., 2011). Notably, the pattern of pubertal development of girls exposed to preeclampsia *in utero* seems to differ from changes in puberty previously reported in girls affected by juvenile obesity born from uncomplicated pregnancies (Ogland et al., 2011). Peripubertal boys and girls exposed to preeclampsia *in utero* have also presented sexually dimorphic alterations in blood concentration of androgens at 11–12 years of age when compared to children born from uncomplicated pregnancies (Alsnes et al., 2016). Importantly, hyperandrogenism has not only been linked to altered female pubertal development, but also to cardiometabolic disorders, including insulin resistance, obesity, hypertension, and preeclampsia (Ogland et al., 2011; Mouritsen et al., 2015; Iwasa et al., 2017; Kumar et al., 2018; Zeng et al., 2020). Nonetheless, the specific pathways in the crosstalk between reproductive and metabolic abnormalities in offspring from preeclamptic mothers remain vastly speculative.

Kisspeptins are a family of small peptides encoded by the *Kiss1* gene with sex-specific roles in reproduction and metabolism (Hussain et al., 2015; Dudek et al., 2018; Harter et al., 2018). Besides the central role of kisspeptins in the activation of the hypothalamic-pituitary-gonadal axis, this family of peptides appear to be important peripheral regulators of metabolism, energy expenditure, thermoregulation and adipogenesis (Hussain et al., 2015; Dudek et al., 2018; Harter et al., 2018; Izzi-Engbeaya et al., 2019; Hudson and Kauffman, 2022; Musa et al., 2022). *In vitro* studies suggest that kisspeptins may induce lipolysis and impair adipocyte glucose uptake in rodents and humans (Hussain et al., 2015; Pruszynska-Oszmalek et al., 2017; Dudek et al., 2018; Hudson and Kauffman, 2022). Interestingly, a sexually dimorphic metabolic phenotype has been reported in mice lacking functional kisspeptin receptor gene (*Kiss1r)* (Tolson et al., 2014; Tolson et al., 2016; Tolson et al., 2019). Namely, global *Kiss1r* knockout (KO) female mice display increased body weight, adiposity, serum leptin concentration and impaired glucose tolerance when compared to wild type littermates. Conversely, global *Kiss1r* KO males have similar body weight and glucose homeostasis as wild type counterparts (Tolson et al., 2014; Tolson et al., 2016; Tolson et al., 2019). Studies in gonadectomized rats suggest that both sex steroid hormones and nutrition modulate adipose tissue *Kiss1* (Brown et al., 2008)*.* Therefore, similar to the central nervous system, we speculate that kisspeptins are also the “missing link” in the crosstalk between reproduction and metabolism in peripheral tissues (Hussain et al., 2015; Dudek et al., 2018; Harter et al., 2018; Izzi-Engbeaya et al., 2019; Hudson and Kauffman, 2022; Musa et al., 2022).

The Blood Pressure High Subline-5 (BPH/5) mouse is a well-established translational model of superimposed preeclampsia (Davisson et al., 2002; Sones et al., 2021). The BPH/5 mouse resulted from an eight-way cross of the mouse strains LP, SJL, BALB/c, C57BL/6, 129, CBA, RF, and BDP, followed by multiple generations of brother-sister matings (Schlager, 1973). BPH/5 females are spontaneously obese, dyslipidemic, hyperleptinemic, hyperphagic and hypertensive, a phenotype exacerbated during pregnancy (Davisson et al., 2002; Reijnders et al., 2019; Sones et al., 2021). Even though long-term adverse outcomes occur in both BPH/5 male and female offspring, a sexually dimorphic cardiometabolic phenotype has been elucidated (Sutton et al., 2017; Beckers et al., 2021). Interestingly, while the obese phenotype is perpetuated in BPH/5 female offspring, BPH/5 males do not present increased food intake, body weight, or reproductive white adipose tissue (rWAT) mass in comparison to controls (Sutton et al., 2017; Beckers et al., 2021). Importantly, the influence of obesity in the preeclamptic-like phenotype of the BPH/5 mouse model has been clearly established (Reijnders et al., 2019; Beckers et al., 2022). Of interest, maternal weight loss *via* a pair-feeding paradigm not only attenuates the BPH/5 mouse maternal inflammatory milieu and pregnancy outcomes, but also improves long-term consequences in the offspring in a sex-specific manner (Reijnders et al., 2019; Beckers et al., 2022).

In this study, the BPH/5 mouse model was utilized to further investigate long-term reproductive outcomes of *in utero* exposure to maternal obesity and a preeclampsia-like syndrome. It was hypothesized that BPH/5 progeny would present abnormal pubertal development when compared to C57BL/6 (C57) control mice. Furthermore, the sexually dimorphic obese phenotype of the BPH/5 mouse was further explored, and a potential link between nutrition, rWAT kisspeptin expression and sex steroid hormone profile was explored, utilizing the well-established BPH/5 pair-feeding paradigm (Reijnders et al., 2019; Beckers et al., 2022).

## 2 Materials and methods

### 2.1 Animal husbandry

Experiments were performed using virgin BPH/5 and control C57 males and females from in-house colonies. The normotensive C57 strain was used in the eight-way cross that originated the BPH/5 (Davisson et al., 2002). Peripubertal (3 weeks of age) and adult (8–12 weeks of age) mice were housed in a climate-controlled environment (12-h light-dark cycle, 70.5–71°F) and fed a standard chow diet (Purina 5001 rodent chow: 23% crude protein, 4.5% crude fat, 6% crude fiber, and 8% ash, Neenah, WI) and *ad libitum* water. For studies using estrous cycle-staged females, vaginal cytology samples were collected daily from single-housed virgin BPH/5 and C57 adult females for at least two complete estrous cycles, in accordance with previous reports, and sample collection was performed during the first day of cytological proestrus (*n* = 3–8/group) (Caligioni, 2009; Cora et al., 2015). In accordance with the American Veterinary Medical Association Guidelines for the Euthanasia of Animals, the animals were humanely euthanized *via* carbon dioxide inhalation, followed by immediate cervical dislocation and exsanguination as secondary methods to ensure death (Leary et al., 2020). All animal procedures were approved by the Institutional Animal Care and Use Committee at Louisiana State University School of Veterinary Medicine and are in accordance with the PHS Guide for the Care and Use of Laboratory Animals.

### 2.2 Assessment of puberty

Peripubertal BPH/5 and C57 males and females were examined daily for signs of puberty starting at 2 weeks of age, utilizing clinical parameters previously described (Novaira et al., 2014). In females, visual assessment of vaginal opening was performed. In males, balanopreputial separation from the glans penis was assessed *via* gentle manual preputial retraction (Supplementary Figures S1B–D). Mice were weaned at 21 days of age, and body weight was recorded using a Gram scale. Post-weaning, littermates were housed in groups by sex. Four BPH/5 and C57 litters were used, with all males and females included in the study. Exclusion criteria included litters with less than four pups at weaning, and male or female mice singly housed post-weaning. Once clinical signs of puberty were noted, namely, vaginal opening or balanopreputial separation, age was recorded, and humane euthanasia was performed. Post-mortem, body weight was recorded, and vaginal opening or balanopreputial separation were confirmed. Testes in males, the female reproductive tract, including the uterus, uterine tubes and ovaries, and rWAT were dissected immediately after euthanasia and wet weights were recorded with an analytical balance (Ohaus EX324N/AD NTEP, Columbia, MD).

### 2.3 Pair-feeding protocol

Weight loss was induced in a cohort of non-pregnant adult BPH/5 females using a feeding paradigm previously established in this mouse model (Reijnders et al., 2019; Beckers et al., 2022). Briefly, the diet was restricted to 3 g of rodent standard chow (Purina 5001, Neenah, WI) per day for a total of 7 days. With this diet, pair-fed BPH/5 females (BPH/5 PF) are expected to consume 25% less calories than ad libitum-fed counterparts (BPH/5 AL), matching the food intake of lean, ad libitum-fed, C57 controls (Reijnders et al., 2019; Beckers et al., 2022). The non-pregnant pair-fed females were humanely euthanized the day after completion of the 7-day diet and sample collection was performed.

### 2.4 Histology

Testicles from adult BPH/5 and C57 mice were fixed in 10% formalin, paraffin embedded, sectioned and stained using hematoxylin and eosin (*n* = 3/group). Tissue architecture was evaluated by a Diplomate of the American College of Veterinary Pathologists that was blinded to the study design. Adipocyte histomorphometry was performed using the Zeiss Zen software (version 3.7), as previously described (Parlee et al., 2014). Specifically, five microscopic fields were randomly selected from each sample/subject by an observer blinded to the study design, and the number of adipocytes per field was calculated. Furthermore, five adipocytes were randomly selected from each microscopic field and the adipocyte diameter was recorded (*n* = 75 adipocytes/group).

### 2.5 Liquid chromatography

Blood was collected from peripubertal and adult BPH/5 and C57 mice *via* cardiac puncture, allowed to clot at room temperature for 60 to 90 min, and centrifuged at 3,500 rpm for 20 min. The serum was collected and cryopreserved at −80°C until further analysis. Serum was submitted for liquid chromatography with tandem mass spectrometry to assess 17β-estradiol and testosterone concentration at the Mayo Clinic Immunochemical Core (Rochester, MN).

### 2.6 Quantitative reverse transcription polymerase chain reaction (qRT-PCR)

Samples of rWAT were collected from adult BPH/5 and C57 mice immediately after humane euthanasia. Aliquots were flash-frozen and cryopreserved at −80°C until further analysis. Genomic DNA was eliminated, and total RNA was extracted using TRIzol according to the manufacturer’s instructions (Thermo Fisher Scientific, Wilmington, United States). The RNA ratio of absorbance and concentration were assessed using a NanoDrop Spectrophotometer (NanoDrop 200, ThermoFisher Scientific, Wilmington, United States) and 1,000 ng cDNA was synthetized using a commercial kit for reverse transcription (qScript cDNA, Quanta Biosciences, Gaithersburg, United States). Quantification of gene expression levels was performed by qRT-PCR using SYBR Green (PerfeCTa SYBR Green FastMix, Quanta Biosciences, Gaithersburg, United States). Each sample was run in triplicates and mRNA expression was normalized to 18 s and analyzed using the ddCT method (Livak and Schmittgen, 2001). Sequence-specific amplification was confirmed by a single peak during the dissociation protocol following amplification and by product size using gel electrophoresis. Gene targets and primer sequences are listed in Supplementary Table S1.

### 2.7 Statistical analysis

Data analyses were performed using GraphPad Prism, version 9.4 (GraphPad Prism Software, Inc., La Jolla, CA, United States). Student’s t-tests were used for comparisons between age- and sex-matched BPH/5 and C57. Welch’s corrections were performed for inequality of variances. One-way ANOVA and *post hoc* Tukey’s test were used for age-matched group comparisons, and two-way ANOVA and *post hoc* Tukey’s test were used for investigations of male mice potential age and strain interactions on testosterone concentration. Furthermore, a simple linear regression was performed between age at vaginal opening and BW within the groups of peripubertal BPH/5 and peripubertal C57 females. Logarithmic transformation was performed for data that did not meet the normality criteria. Normality of residuals from the models were accessed and confirmed *via* Shapiro-Wilk tests. Data are presented as mean ± SEM. Significance was set at *p* < 0.05.

## 3 Results

### 3.1 Pubertal development is altered in the preeclamptic-like BPH/5 mouse offspring in a sex-dependent manner

**FIGURE 1:**
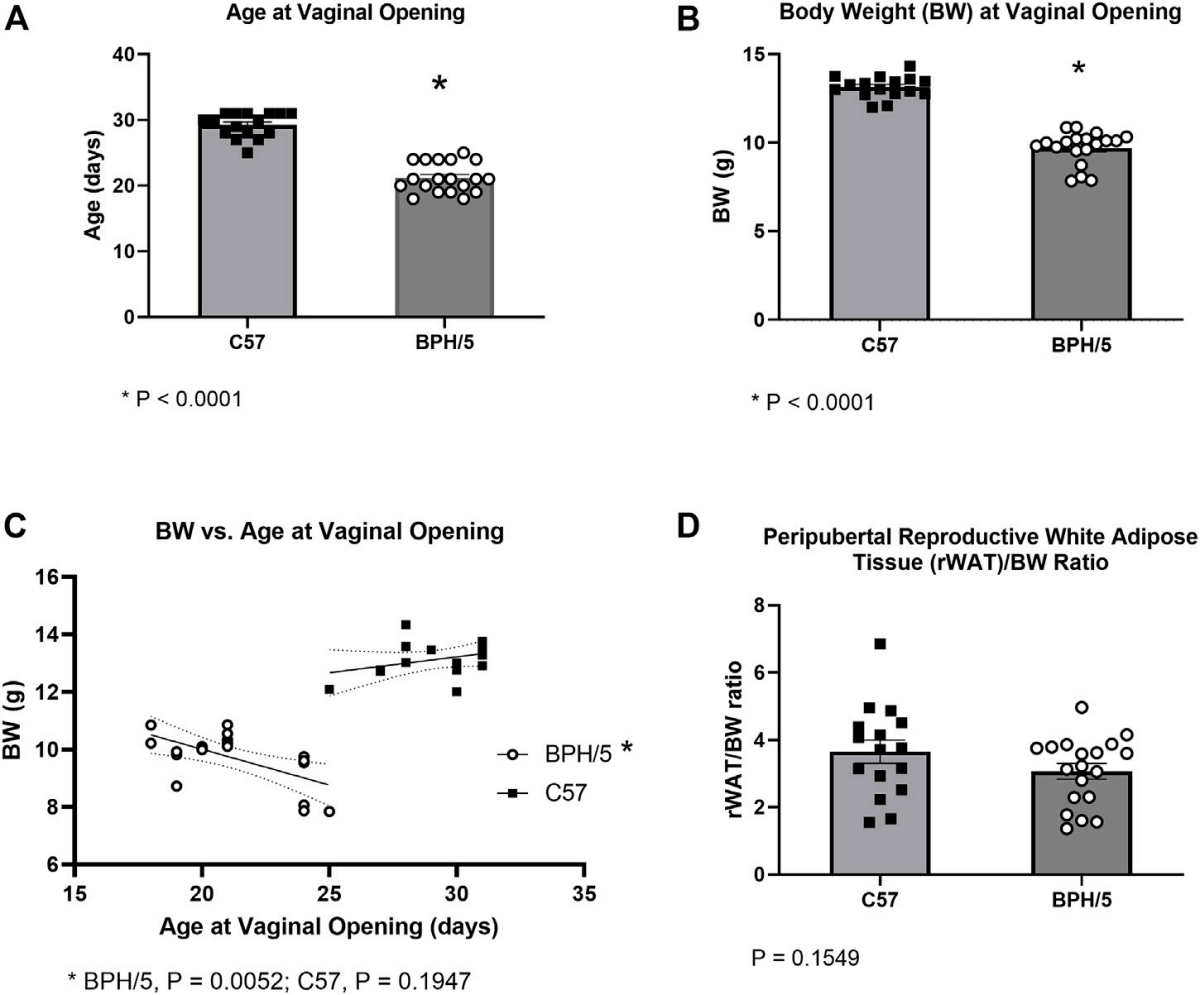
Precocious pubertal development occurs in the preeclamptic-like BPH/5 female offspring. **(A)** Age at the day of vaginal opening in BPH/5 and C57 female offspring (*n* = 16–19/group, Student’s t-test, mean ± SEM, *p < 0.0001). **(B)** Body weight (BW) at the day of vaginal opening in peripubertal BPH/5 and C57 female offspring (*n* = 16–19/group, Student’s t-test, mean ± SEM, *p < 0.0001). **(C)** A negative correlation occurs between BW and age at vaginal opening in BPH/5 females (*n* = 19, Simple linear regression with 95% confidence bands of best-fit line, *p* = 0.0052), while a significant correlation was not seen in C57 controls (*n* = 16, Simple linear regression with 95% confidence bands of best-fit line, *p* = 0.1974). **(D)** Reproductive white adipose tissue weight corrected to BW (rWAT/BW ratio) in peripubertal BPH/5 and C57 females at the day of vaginal opening (*n* = 16–19/group, Student’s t-test, mean ± SEM, *p* = 0.1549).

A total of four BPH/5 and four C57 litters were included in the cohort utilized for peripubertal investigations. Since one C57 litter contained a single male, only the females from that litter were included in further studies. Age at the time of vaginal opening (females) and balanopreputial separation (males) were used as clinical signs of onset of puberty. The BPH/5 female offspring presented precocious pubertal development in comparison to C57 counterparts. Mean age at vaginal opening was 21.21 ± 2.28 days in BPH/5 females (mean ± SD), compared to 29.25 ± 1.88 days in C57 females (Figure 1A, *p* < 0.0001). Since age at weaning was fixed at 21 days, many BPH/5 females achieved puberty before weaning, while pre-weaning vaginal opening was not observed in C57 progeny. Body weight and adiposity were further investigated in peripubertal BPH/5 females. At the onset of puberty, BPH/5 females had lower body weight than C57 controls (Figure 1B, *p* < 0.0001). While a negative correlation between body weight and age at vaginal opening occurred in BPH/5 females (Figure 1C, *p* = 0.0052), a significant correlation was not observed in the C57 group (Figure 1C, *p* = 0.1947). Remarkably, despite the lower body weight of BPH/5 females during onset of puberty, the corrected rWAT mass (rWAT/body weight ratio) was not different between BPH/5 and C57 at the day of vaginal opening (Figure 1D, *p* = 0.1549).

**FIGURE 2:**
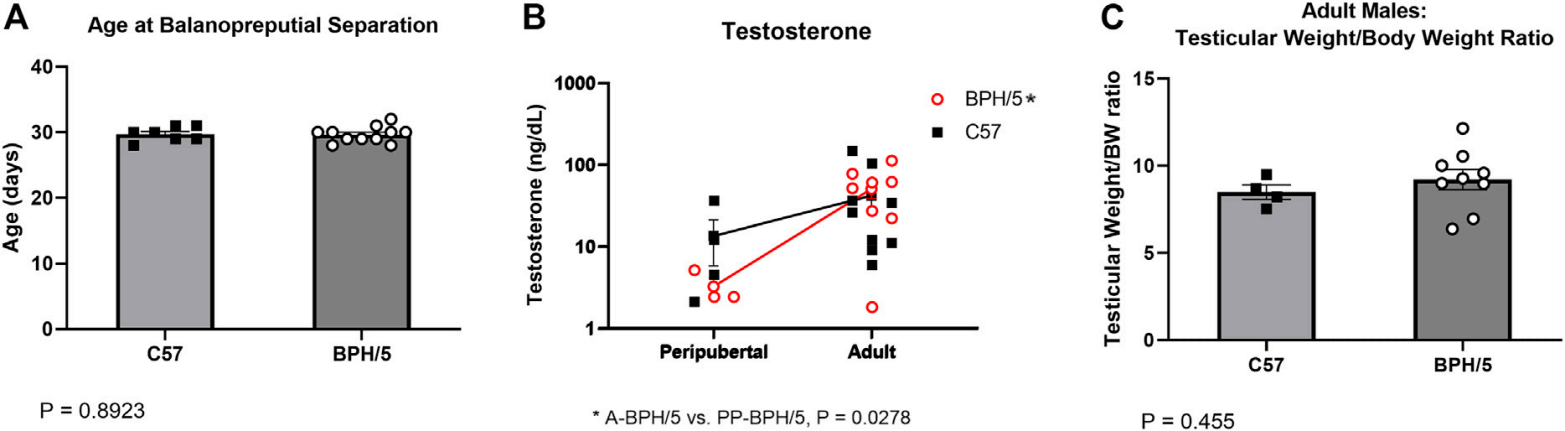
Precocious pubertal development does not occur in BPH/5 male offspring. **(A)** Age at the day of balanopreputial separation in peripubertal BPH/5 and C57 male offspring (*n* = 7–11/group, Student’s t-test, mean ± SEM, *p* = 0.8923). **(B)** Serum testosterone concentration in peripubertal and adult BPH/5 and C57 males (*n* = 3–9/group, Two-way ANOVA, *post hoc* Tukey’s test, mean ± SEM, *p = 0.0278 in adult BPH/5 vs. peripubertal BPH/5). **(C)** Combined testicular weight (i.e., both testes) corrected by body weight (Testicular weight/BW ratio) in adult BPH/5 males and age matched C57 (*n* = 8–17/group, Student’s t-test, mean ± SEM, *p* = 0.455).

In males, age at balanopreputial separation, a testosterone-dependent event, was not different between BPH/5 and C57, with mean ages of 29.64 ± 1.20 and 29.71 ± 1.11 days, respectively (Figure 2A, *p* = 0.8923). Accordingly, serum testosterone concentration was not different between age matched BPH/5 vs. C57 males, ranging from 2.1 (min) to 36 (max) ng/dL (median = 4.5 ng/dL) in peripubertal animals, and 1.8 (min) to 146 ng/dL (median = 34.0 ng/dL) in adults (Figure 2B, *p* > 0.05). Non-etheless, testosterone concentration was significantly higher in adult BPH/5 males when compared to peripubertal BPH/5 (*p* = 0.0278), but not in adult C57 males when compared to peripubertal C57 (*p* = 0.3849). Corrected testicular weight was not different between adult BPH/5 and C57 males, and histopathological abnormalities were not noted (Figure 2C, *p* = 0.455).

### 3.2 Female BPH/5 offspring present hyperandrogenism during adulthood, which is attenuated by dietary weight loss

**FIGURE 3:**
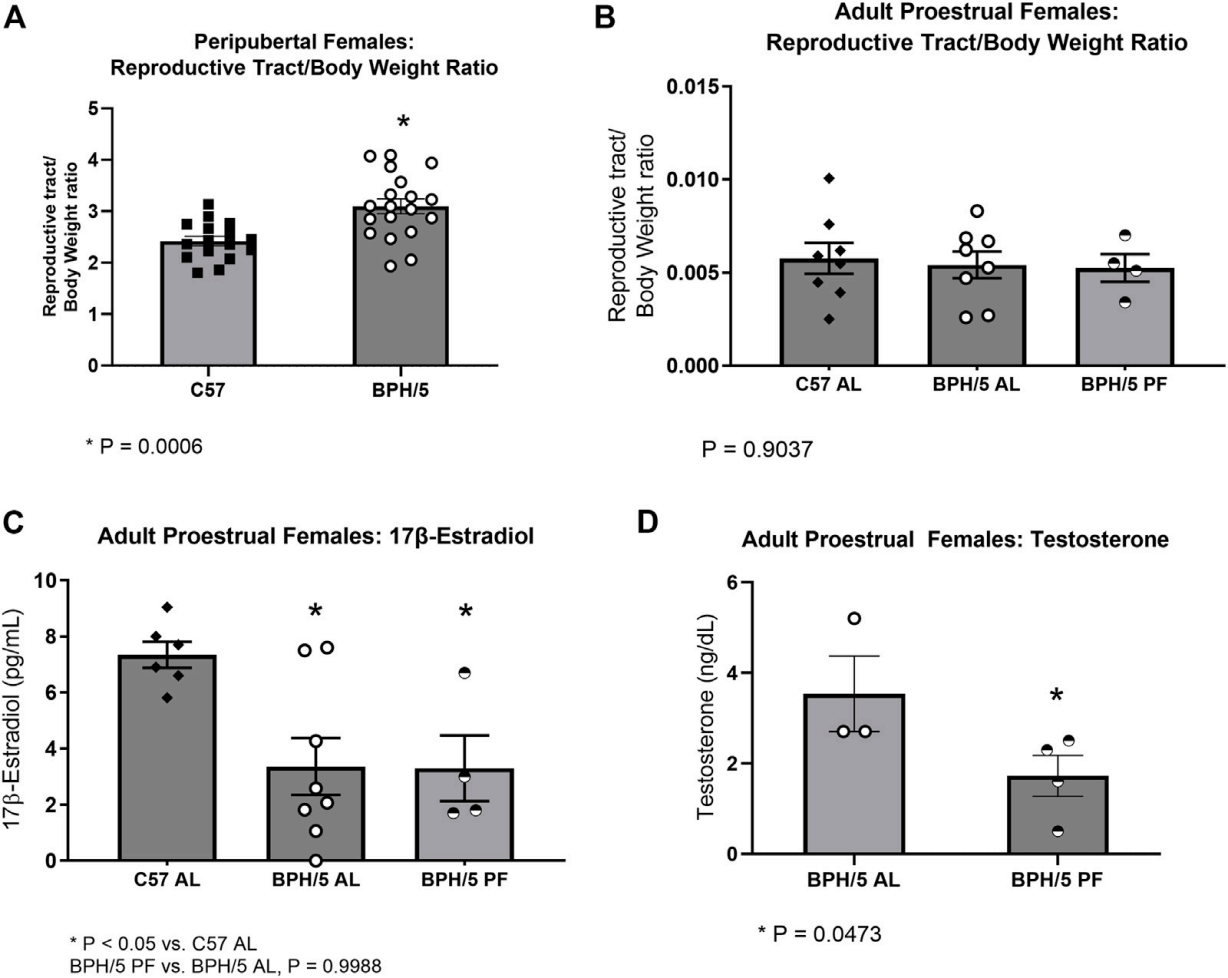
Female BPH/5 offspring present hyperandrogenism during adulthood, which is attenuated by dietary weight loss. **(A)** Reproductive tract wet weight corrected to body weight (BW) in peripubertal BPH/5 and C57 females (*n* = 16–19/group, Student’s t-test, mean ± SEM, *p = 0.0006). **(B,C)** As previously reported (Sutton et al., 2017), adult ad libitum-fed BPH/5 females (BPH/5 AL) present lower serum 17β-estradiol and similar uterine wet weight to ad libitum-fed C57 (C57 AL) during proestrus. In this study, **(B)** reproductive tract/body weight ratio and **(C)** serum 17β-estradiol were unchanged in proestrual pair-fed BPH/5 females (BPH/5 PF) vs. BPH/5 AL females (*n* = 4–8/group, One-way ANOVA and *post hoc* Tukey’s test, mean ± SEM, *p < 0.05 vs. C57 AL). **(D)** Serum testosterone concentration was below the assay detection limit of 2 ng/dL in proestrual C57 AL. Serum testosterone concentration was detected in proestrual BPH/5 AL females and was attenuated in BPH/5 PF females (*n* = 3–4/group, Student’s t-test, mean ± SEM, *p = 0.0473).

It has been previously shown that adult BPH/5 females present aberrant estrous cycles and decreased 17β-estradiol during proestrus (Sutton et al., 2017). Additionally, adult BPH/5 females present increased uterine wet weight during diestrus, which may be associated with tissue inflammation (Sutton et al., 2017). Interestingly, peripubertal BPH/5 females also present a higher reproductive tract wet weight at the day of vaginal opening (Figure 3A, *p* = 0.0006). To further characterize the endocrine profile of BPH/5 offspring, serum concentration of 17β-estradiol was investigated in peripubertal animals, but was below the assay detection limit (3 pg/mL) in all peripubertal BPH/5 and C57 females investigated. In addition to estrogens, androgens have an important role in the sex steroid hormone control of female pubertal development. There is also compelling evidence of a link between hyperandrogenism and metabolic disorders such as hyperphagia and obesity (Iwasa et al., 2017; Leeners et al., 2017). Similar to 17β-estradiol, however, serum testosterone concentration was below the assay detection limit (2 ng/dL) in all peripubertal animals studied.

We have previously shown attenuation of the obesogenic and inflammatory phenotype of BPH/5 females *via* dietary weight loss by either one or 2 weeks of pair-feeding to C57 counterparts (Sutton et al., 2017; Reijnders et al., 2019). Curiously, pair feeding adult BPH/5 females appear to be associated with regularization of estrual cyclicity. Namely, while irregular estrous cycles are commonly seen in ad libitum-fed BPH/5 females, approximately 80% of pair-fed BPH/5 females present regular 4–5 days cycles. Herein, the influence of dietary weight loss in the BPH/5 female sex steroid hormone profile was investigated. In accordance with previous reports, reproductive tract wet weight/body weight ratio was not different between ad libitum-fed BPH/5 and C57 females during proestrus and was not changed by dietary weight loss (Sutton et al., 2017) (Figure 3B, *p* = 0.9037). Likewise, hypoestrogenism was unchanged by controlled food intake. Specifically, the previously described lower serum 17β-estradiol concentration in BPH/5 during proestrus (Figure 3C, *p* < 0.05) was not altered by 7 days of pair feeding (Sutton et al., 2017) (Figure 3C, *p* = 0.9988). Interestingly, however, while serum testosterone concentration was lower than the assay detection limit (2 ng/dL) in adult C57 females, it ranged from 2.7 (min) to 5.2 (max) ng/dL (median = 2.7 ng/dL) in adult BPH/5 females during proestrus and was attenuated by 1 week of pair feeding (Figure 3D, *p* = 0.0473).

### 3.3 Sexually dimorphic adipocyte hypertrophy and altered kisspeptin/receptor expression occurs in reproductive white adipose tissue of adult BPH/5 offspring

**FIGURE 4:**
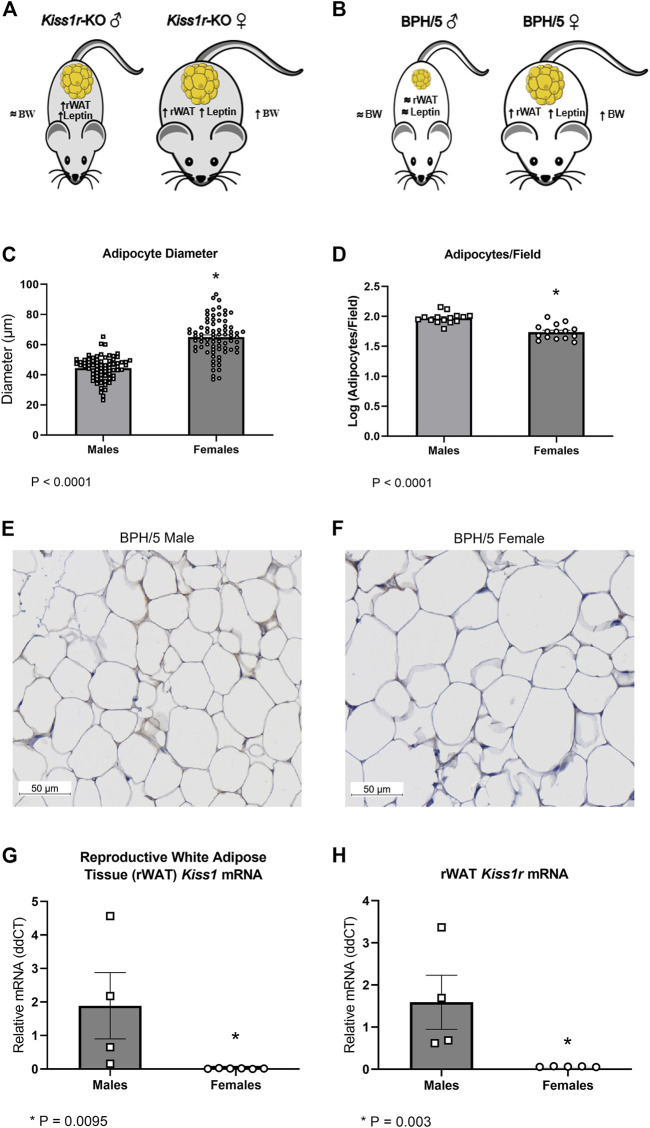
Sexually dimorphic adipocyte hypertrophy and altered kisspeptin/receptor expression occurs in reproductive white adipose tissue of adult BPH/5 offspring. **(A)** Previously described sexually dimorphic metabolic phenotype global *Kiss1r* knockout (KO) mice (Tolson et al., 2014; Tolson et al., 2016). **(B)** Sexually dimorphic metabolic phenotype of adult BPH/5 offspring. **(C)** Average adipocyte diameter (µm) in the reproductive white adipose tissue (rWAT) of BPH/5 males and BPH/5 females (*n* = 75 adipocytes/group, Student’s t-test, mean ± SEM, *p < 0.0001). **(D)** Number of adipocytes per microscopic field in the rWAT of adult BPH/5 males and females (*n* = 15 fields/group, Student’s t-test, mean ± SEM, *p < 0.0001). **(E,F)** Representative photomicrographs of rWAT of adult **(E)** BPH/5 males and **(F)** BPH/5 females (scale bar = 50 µm). **(G)** *Kiss1* relative mRNA expression in rWAT of adult BPH/5 males and females (*n* = 4–6/group, Student’s t-test, mean ± SEM, *p = 0.0095). **(H)** *Kiss1r* relative mRNA expression in rWAT of adult BPH/5 males and females (*n* = 4–6/group, Student’s t-test, mean ± SEM, *p = 0.003).

**FIGURE 5:**
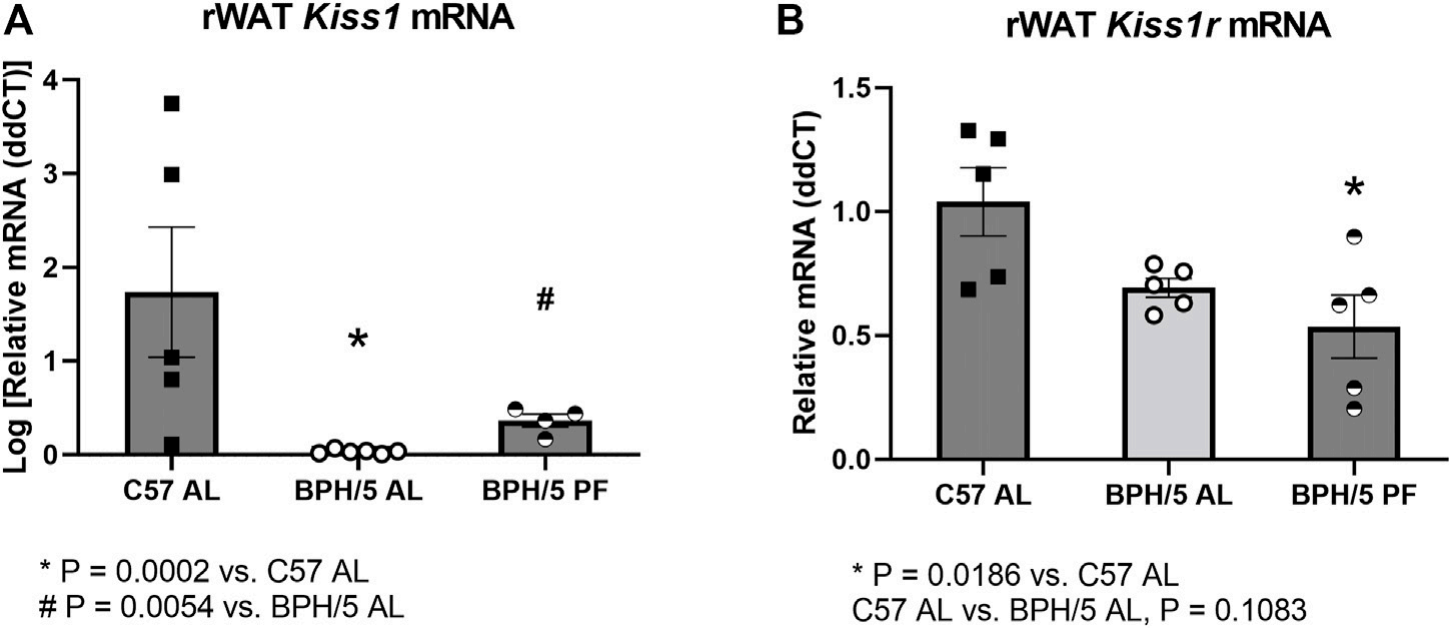
Reproductive white adipose tissue kisspeptin downregulation is ameliorated in BPH/5 adult females by dietary weight loss. **(A)** *Kiss1* is downregulated in ad libitum-fed adult BPH/5 female (BPH/5 AL) rWAT when compared to C57 AL. The downregulation is attenuated in BPH/5 female rWAT by pair feeding (BPH/5 PF) to mirror C57 AL food intake (*n* = 4–6/group, One-way ANOVA and post-hoc Tukey’s test, mean ± SEM, *p = 0.0002 vs. C57 AL, # *p* = 0.0054 vs. BPH/5 AL). **(B)** rWAT *Kiss1r* expression was not different between BPH/5 AL and C57 AL, while rWAT *Kiss1r* was lower in BPH/5 PF when compared to C57 AL (*n* = 5/group, One-way ANOVA and post-hoc Tukey’s test, mean ± SEM, *p = 0.0186 vs. C57 AL, *p* = 0.1083 in BPH/5 AL vs. C57 AL.

Striking phenotypic similarities exist between the previously described global *Kiss1r* KO mice (Figure 4A) and the BPH/5 mouse model (Figure 4B), in a sex-dependent manner (Tolson et al., 2014; Tolson et al., 2016; Tolson et al., 2019). Specifically, adult BPH/5 females present markedly increased body weight, adiposity and serum leptin concentration in comparison to adult C57 females. Conversely, adult BPH/5 males have similar body weight than their control counterparts, adult C57 males. When investigating BPH/5 rWAT histomorphometry, adult BPH/5 females displayed increased mean adipocyte diameter when compared to males (Figure 4C, *p* < 0.0001) and, consequently, a lower number of adipocytes per microscopic field (Figure 4D, *p* < 0.0001), findings suggestive of adipocyte hypertrophy in females (Figures 4E, F) (Parlee et al., 2014). Considering the potential role of kisspeptins in adipocyte function, the expression of *Kiss1* and *Kiss1r* in the rWAT of BPH/5 male and female offspring was investigated. Both *Kiss1* and *Kiss1r* were downregulated in the rWAT of adult BPH/5 females when compared to age-matched males (Figures 4G, H, *p* = 0.0095 and 0.003, respectively). Of note, *Kiss1* downregulation was also seen in BPH/5 females when compared to sex- and age-matched C57 controls (Figure 5A, *p* = 0.0002), while rWAT *Kiss1* and *Kiss1r* were not different between adult BPH/5 and A-C57 males (Supplementary Figure 2, *p* > 0.05).

### 3.4 Reproductive white adipose tissue kisspeptin downregulation is ameliorated in BPH/5 females by dietary weight loss

While there is evidence that kisspeptins may function as regulators of lipolysis and adipogenesis, particularly in females, the upstream mechanisms modulating rWAT *Kiss1* expression remain largely unknown. The expression of *Kiss1*/*Kiss1r* seems to be directly associated with the sex steroid hormone milieu in the brain and multiple peripheral tissues, including the adipose tissue in gonadectomized rats, and the uterus and placenta of the BPH/5 mouse model (Hou and Gorski, 1993; Brown et al., 2008; Cejudo Roman et al., 2012; Baba et al., 2015; Hussain et al., 2015; Stephens et al., 2015; Dudek et al., 2018; Harter et al., 2018; Schaefer et al., 2021; Gomes et al., 2022). However, there is also evidence of a direct role of nutritional status on adipose tissue *Kiss1* expression, with upregulation promoted by high fat diet, and downregulation promoted by fasting (Brown et al., 2008). Therefore, the rWAT *Kiss1/Kiss1r* was also investigated in adult BPH/5 females subjected to 7 days of pair feeding to age- and sex-matched C57. In agreement with previous findings, rWAT *Kiss1* downregulation was attenuated in pair-fed BPH/5 females (BPH/5 PF), when compared to ad libitum-fed BPH/5 counterparts (BPH/5 AL, Figure 5A, *p* = 0.0054). While rWAT *Kiss1r* was not significantly lower in BPH/5 AL vs. C57 AL (Figure 5B, *p* = 0.1083), it was lower in BPH/5 PF females when compared to C57 AL (Figure 5B, *p* = 0.0186).

## 4 Discussion

In this study, the BPH/5 mouse model was used to further investigate transgenerational reproductive and metabolic outcomes of a preeclampsia-like syndrome. Herein, we provide evidence of sex-specific abnormalities of pubertal development in offspring prenatally exposed to an obesogenic and hypertensive uterine environment. In accordance with previous studies, the day of vaginal opening and balanopreputial separation were selected as clinical signs of onset of puberty (Lapatto et al., 2007; Caligioni, 2009; Novaira et al., 2014). While BPH/5 female offspring presented marked precocious pubertal development, age at balanopreputial separation was not different between BPH/5 male offspring and C57 controls. Accordingly, serum testosterone concentration and testicular wet weight were also similar between age matched BPH/5 and C57 males. As one could anticipate based on their younger age, BPH/5 females had lower body weight than C57 at the day of vaginal opening. Notably, however, peripubertal BPH/5 females presented similar rWAT/body weight ratio as age-matched C57, further highlighting the role of adiposity in pubertal development.

The correlation between body weight, body composition and puberty has long been recognized (Baker, 1985). Specifically, a minimum body weight and fat mass are required for onset and progression of pubertal development (Baker, 1985; Rosenfield et al., 2009). In peripubertal girls, normal age at onset of puberty ranges from 8–13 years old, and the normal progression of pubertal development is breast development, termed thelarche, followed by axillary and pubic hair growth (pubarche) and onset of menses (menarche) (Rosenfield et al., 2009; Ogland et al., 2011). There is a rising incidence of early onset of puberty in girls, which has been closely associated to juvenile obesity (Jasik and Lustig, 2008; Rosenfield et al., 2009). Notably, however, while obesity *per se* is associated with hastened thelarche, daughters from preeclamptic pregnancies may present an abnormal progression of pubertal development, with pubarche preceding thelarche, particularly in offspring from severe preeclampsia (Jasik and Lustig, 2008; Ogland et al., 2011). To date, the mechanisms and clinical significance of those findings are not known.

An adverse intrauterine environment has an important role in postnatal offspring outcomes of preeclampsia. Prenatal starvation and fetal growth restriction may lead to altered *in utero* metabolic programing, excessive compensatory growth, obesity, and adverse cardiovascular outcomes later in life (Jain and Singhal, 2012; Jensen et al., 2015). Correspondingly, children born from preeclamptic pregnancies often have higher BMI postnatally, and higher risk of developing obesity (Ogland et al., 2011; Yang et al., 2021). High rate of BMI increase from 6 to 12 years of age has been associated with increased risk of cardiovascular disease (Yuan et al., 2020; Yang et al., 2021). Additionally, the risk of preeclampsia is reportedly 3-fold higher in obese women (i.e., body mass index >30 kg/m^2^) when compared to lean counterparts (Mbah et al., 2010). Since daughters from preeclamptic women are more prone to develop preeclampsia (Arngrimsson et al., 1990), it is speculated that fetal growth restriction, excessive compensatory growth and obesity in girls born from preeclamptic mothers may contribute to self-perpetuation of this syndrome.

Adverse fetal programing seems to be recapitulated by the preeclamptic-like BPH/5 mouse model. When reciprocal breeding crosses of BPH/5 and C57 pairs were performed, only embryos gestated in BPH/5 dams presented delayed embryonic development (Sones et al., 2016). Additionally, BPH/5 offspring are affected by intrauterine growth restriction, evidenced by fetal demise, smaller litter sizes, and marked decrease in birth weight when compared to C57 controls (Davisson et al., 2002). Postnatally, excessive compensatory growth from birth to early adulthood has been reported in BPH/5 females, but not males (Sutton et al., 2017; Beckers et al., 2021). Specifically, while BPH/5 females are smaller than C57 controls at postnatal day 1, no difference in body weight is seen at 3 weeks of age, and higher body weight is displayed by 8-week-old BPH/5 females (Sutton et al., 2017). It is therefore speculated that higher growth rate and adiposity in BPH/5 females from birth to early adulthood may trigger earlier onset of puberty. Leptin, a metabolic hormone mainly derived from white adipose tissue and placenta, has an important role in metabolism and pubertal development, providing cues to the hypothalamic-pituitary-gonadal axis (Kiess et al., 1999; Messager et al., 2005; De Bond et al., 2016). Hyperleptinemia has been demonstrated in non-pregnant and pregnant BPH/5 females, but does not seem to occur in males, which is in agreement with the sexually dimorphic reproductive phenotype reported herein (Sutton et al., 2017; Reijnders et al., 2019; Beckers et al., 2021). Nonetheless, further investigation of serum leptin concentration and hypothalamic leptin signaling in the peripubertal BPH/5 mouse is warranted.

Hypoestrogenism and hyperandrogenism have been described in women carrying preeclamptic pregnancies and in daughters gestated in a preeclamptic uterus (Ogland et al., 2011; Alsnes et al., 2016; Berkane et al., 2018; Kumar et al., 2018; Keya et al., 2019). A protective metabolic role of estrogens in females is widely recognized, with low estrogens directly associated with increased body weight and adiposity (Leeners et al., 2017). Notably, high testosterone levels in females may further disrupt mechanisms that prevent hyperphagia and adiposity, leading to exacerbated food intake, leptin resistance, and obesity (Iwasa et al., 2017; Leeners et al., 2017). Additionally, hyperandrogenism seems to be associated with pubarche superseding thelarche in girls born from preeclamptic women (Mouritsen et al., 2015; Alsnes et al., 2016). Although 17β-estradiol and testosterone were below the assay detection limit in peripubertal females in this study, BPH/5 females presented higher reproductive tract/body weight ratios at the day of vaginal opening. It remains to be determined if the increased reproductive tract weight of peripubertal BPH/5 females is due to a sex steroid hormone imbalance or uterine inflammation, as suspected in adult BPH/5 (Sutton et al., 2017). Considering the previously reported irregular estrous cyclicity of adult BPH/5 females and hypoestrogenism during proestrus, this estrous cycle stage was selected for further sex steroid hormone profile characterization in BPH/5 female offspring (Sutton et al., 2017). Interestingly, hyperandrogenism accompanies hypoestrogenism in proestrual BPH/5 mice. The source of excessive androgens in girls born from preeclamptic mothers and in preeclamptic-like BPH/5 females is yet to be fully elucidated. Abnormal in-utero programing of steroidogenic enzyme activity, particularly aromatase, the rate-limiting enzyme in the conversion of androgens to estrogens, is speculated in preeclamptic offspring, and may also occur in the BPH/5 mouse model (Mouritsen et al., 2015; Alsnes et al., 2016; Berkane et al., 2018; Noyola-Martinez et al., 2019). Importantly, a metabolic-endocrine interplay is emphasized by the attenuation of hyperandrogenism in proestrual BPH/5 females after 1 week of dietary restriction.

Kisspeptins are considered the “gatekeepers” of the hypothalamic-pituitary-gonadal axis, since hypothalamic kisspeptin signaling is critical for pubertal development and reproduction in males and females (de Roux et al., 2003; Funes et al., 2003; Seminara et al., 2003; Lapatto et al., 2007; Tassigny et al., 2007). Besides their role in the central nervous system, kisspeptins seem to have important roles in peripheral metabolic tissues, including sex-specific regulation of adiposity (Hussain et al., 2015; Dudek et al., 2018; Wang et al., 2018; Izzi-Engbeaya et al., 2019; Hudson and Kauffman, 2022). Altered adipose tissue *Kiss1* expression has been previously associated with obesity in rats and humans (Brown et al., 2008; Cockwell et al., 2013). Furthermore, studies using *Kiss1r* KO mice have shown that impaired kisspeptin signaling leads to increased body weight, adiposity, and hyperleptinemia in adult female mice, along with decreased energy expenditure and impaired glucose tolerance (Tolson et al., 2014; Tolson et al., 2016; Tolson et al., 2019). Conversely, *Kiss1r* KO males display normal body weight and glucose homeostasis when compared to WT littermates (Tolson et al., 2014; Tolson et al., 2019). Herein, we have shown a sexually dimorphic *Kiss1/Kiss1r* dysregulation in the rWAT of preeclamptic-like BPH/5 offspring and hypothesize that rWAT kisspeptin downregulation is linked to obesity in BPH/5 females.

A series of *in vitro* studies using mouse (3T3-L1) and rat adipocytes have shown that kisspeptin-10 inhibits cell proliferation and viability, reduces the intensity of intracellular glucose uptake and triglyceride synthesis, and stimulates basal lipolysis (Pruszynska-Oszmalek et al., 2017). Histomorphometry of adult BPH/5 rWAT is suggestive of adipocyte hypertrophy in females when compared to males (Parlee et al., 2014). Further mechanistic investigations are warranted to confirm if lower levels of kisspeptin-10 in BPH/5 female offspring are associated with adipocyte glucose uptake, lipogenesis, and adipocyte engorgement. Although the upstream regulators of adipose tissue kisspeptin expression are poorly understood, studies suggest that adipose tissue Kiss*1* expression is influenced by nutrition and the sex steroid hormone milieu (Brown et al., 2008). Namely, exogenous administration of testosterone and 17β-estradiol to gonadectomized male and female rats, respectively, increased adipose tissue *Kiss1* expression (Brown et al., 2008). Additionally, while 6 h of fasting led to adipose tissue *Kiss1* upregulation, *Kiss1* downregulation occurred in rats after 19 days of high fat diet (Brown et al., 2008). Accordingly, both serum testosterone and rWAT *Kiss1* were ameliorated in a cohort of BPH/5 females subjected to dietary weight loss compared to ad libitum-fed counterparts. Hence, we speculate that rWAT *Kiss1* downregulation in BPH/5 females may result from an abnormal sex steroid hormone profile from puberty onward, somewhat “closing the circle” between reproduction and adipose tissue regulation in a preeclampsia-like syndrome. While mice globally lacking either functional *Kiss1* or *Kiss1r* have impaired pubertal development (i.e., hypogonadotropic hypogonadism), the BPH/5 mouse may be a suitable additional model to further study the crosstalk between reproductive hormones, adipose tissue kisspeptin dysregulation and adiposity (de Roux et al., 2003; Funes et al., 2003; Seminara et al., 2003; Tassigny et al., 2007; Tolson et al., 2019).

•In summary, beyond the long-term adverse cardiometabolic outcomes, offspring born to obese and preeclamptic-like BPH/5 dams also display sex-specific reproductive abnormalities during pubertal development and early adulthood. In BPH/5 female offspring, the crosstalk between metabolic and reproductive abnormalities seem to involve altered levels of sex steroid hormones and adipose tissue kisspeptin signaling, which may be consequences of abnormal fetal programming. Excitingly, the preeclamptic-like BPH/5 mouse model closely recapitulates clinical findings of children exposed to a hypertensive uterine environment and may be a suitable model to unravel the mechanisms underlying this altered phenotype.

## Data Availability

The original contributions presented in the study are included in the article/Supplementary Materials, further inquiries can be directed to the corresponding author.
