# Supplementary material for: Sexually dimorphic pubertal development and adipose tissue kisspeptin dysregulation in the obese and preeclamptic-like BPH/5 mouse model offspring
Source: Front Physiol. 2023 Mar 23;14:1070426. doi: 10.3389/fphys.2023.1070426 (PMC10076539; doi:10.3389/fphys.2023.1070426)
Supplement: Supplementary file 1 [file Table1.DOCX]

Supplementary Material

# Supplementary Table

Forward and reverse *Mus musculus*-specific primer sequences used in qRT-PCR

| Gene | Primer Sequence | Reference |
| --- | --- | --- |
| *18S* | F: 5’GTAACCCGTTGAACCCCATT-3’; R: 5’CCATCCAATCGGTAGTAGCG3’ | (Sones et al., 2016) |
| *Kiss1* | F: 5' CGAAGGAGTTCCAGTTGTAGG3'  R: 5'AAGGAATCGCGGTATGCA3' | (Zhang et al., 2014) |
| *Kiss1r* | F: 5'CCGTCCAACGCTTCAGGAT3'  R: 5'GTGTAGCGAAAAACAGGGGAA3' | (Zhang et al., 2014) |

#
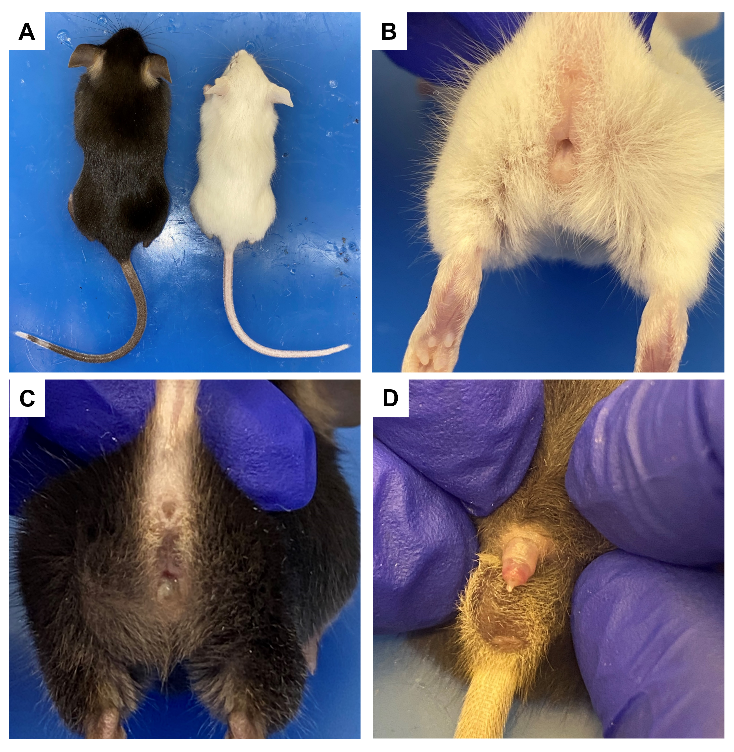
Supplementary Figures

**Supplement Figure 1:** (A) Comparison of body size between C57 (left) and BPH/5 (right) females at the day of vaginal opening. Representative picture of vaginal opening in (B) BPH/5 and (C) C57 females. (D) Representative picture of complete balanopreputial separation in C57 male.


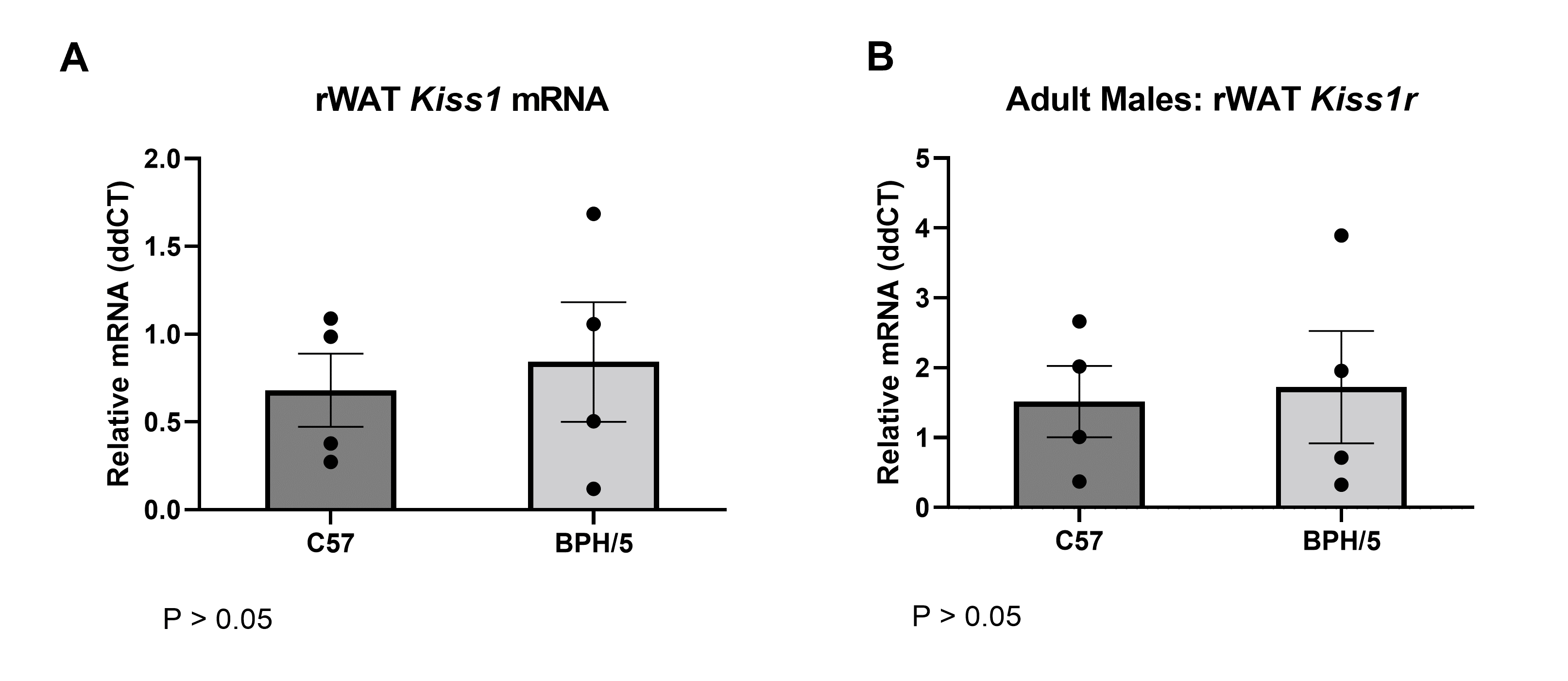


**Supplement Figure 2:** Reproductive white adipose tissue (rWAT) expression of the kisspeptin-encoding gene (*Kiss1)* and the cognate kisspeptin receptor (*Kiss1r)* in adult BPH/5 males and C57 males. Both *Kiss1* (A) and *Kiss1r* (B) were not different in the rWAT of BPH/5 and C57 males (n = 4/group, Student’s t-test, mean ± SEM, P > 0.05 vs. C57 males).

# Supplementary References

Sones, J.L., Cha, J., Woods, A.K., Bartos, A., Heyward, C.Y., Lob, H.E., et al. (2016). Decidual Cox2 inhibition improves fetal and maternal outcomes in a preeclampsia-like mouse model. *JCI Insight* 1(3). doi: 10.1172/jci.insight.75351.

Zhang, P., Tang, M., Zhong, T., Lin, Y., Zong, T., Zhong, C., et al. (2014). Expression and function of kisspeptin during mouse decidualization. *PLoS One* 9(5)**,** e97647. doi: 10.1371/journal.pone.0097647.
