# Supplementary figures and images for: Sexually dimorphic pubertal development and adipose tissue kisspeptin dysregulation in the obese and preeclamptic-like BPH/5 mouse model offspring
Source: Front Physiol. 2023 Mar 23;14:1070426. doi: 10.3389/fphys.2023.1070426 (PMC10076539; doi:10.3389/fphys.2023.1070426)

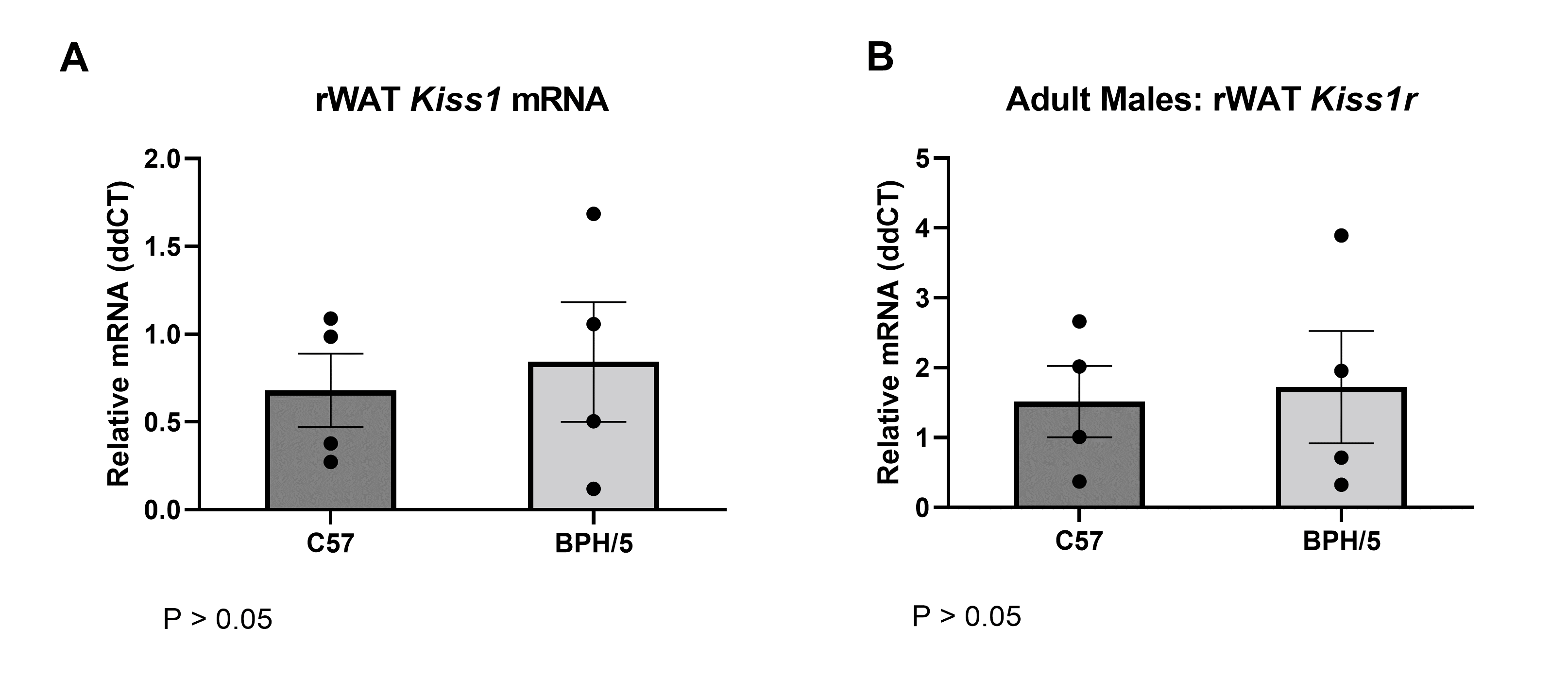

Supplement: Supplementary file 2 [file Image2.tif]

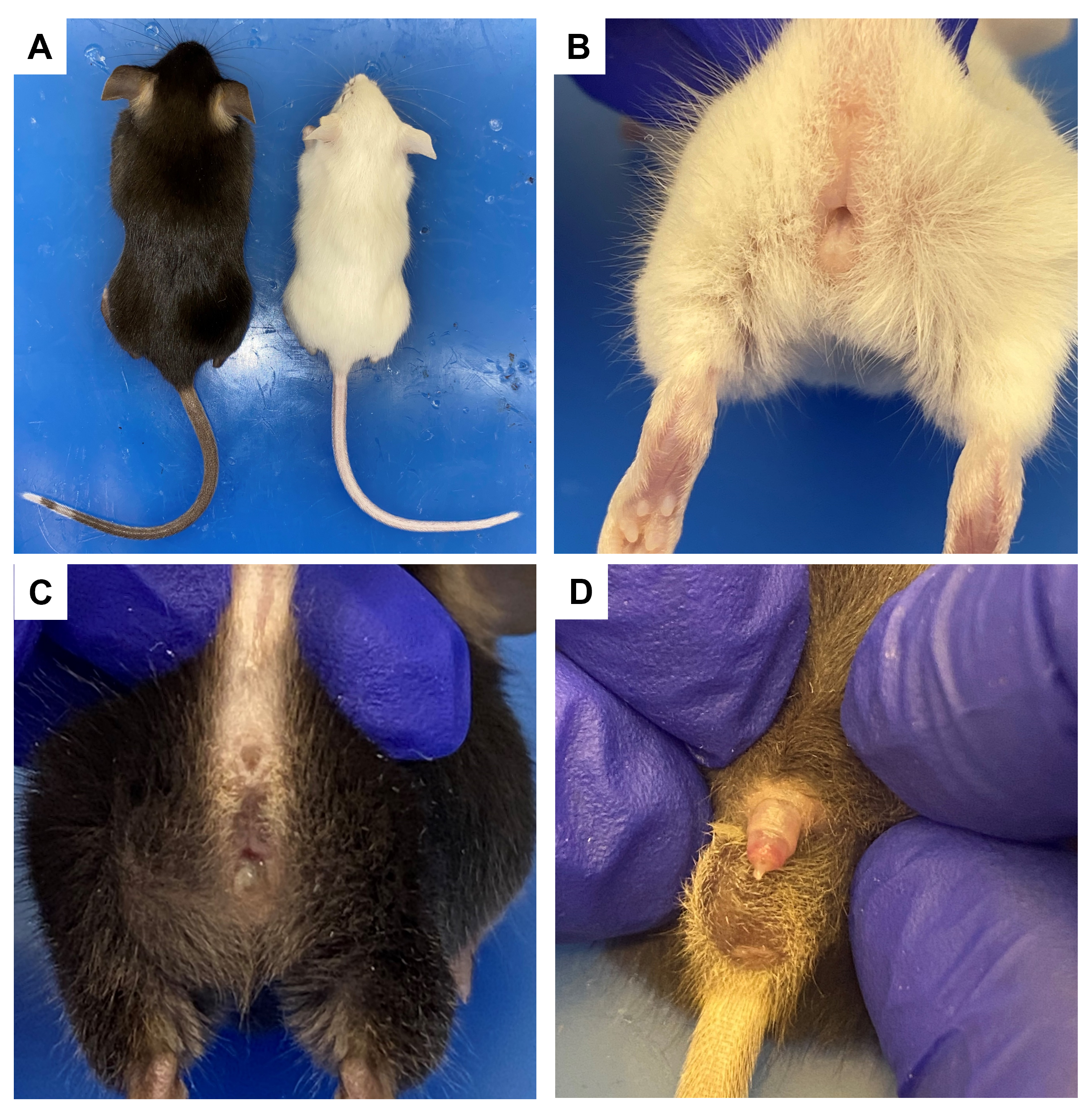

Supplement: Supplementary file 3 [file Image1.tif]
